# Circulating MicroRNA Responses between ‘High’ and ‘Low’ Responders to a 16-Wk Diet and Exercise Weight Loss Intervention

**DOI:** 10.1371/journal.pone.0152545

**Published:** 2016-04-21

**Authors:** Evelyn B. Parr, Donny M. Camera, Louise M. Burke, Stuart M. Phillips, Vernon G. Coffey, John A. Hawley

**Affiliations:** 1 Mary MacKillop Institute for Health Research, Centre for Exercise and Nutrition, Australian Catholic University, Melbourne, Australia; 2 Department of Sports Nutrition, Australian Institute of Sport, Canberra, Australia; 3 Exercise Metabolism Research Group, Department of Kinesiology, McMaster University, Hamilton, Canada; 4 Bond Institute of Health and Sport and Faculty of Health Sciences and Medicine, Bond University, Gold Coast, Queensland, Australia; 5 Research Institute for Sport and Exercise Sciences, Liverpool John Moores University, Liverpool, United Kingdom; Indiana University Richard M. Fairbanks School of Public Health, UNITED STATES

## Abstract

**Background:**

Interactions between diet, physical activity and genetic predisposition contribute to variable body mass changes observed in response to weight loss interventions. Circulating microRNAs (c-miRNAs) may act as ‘biomarkers’ that are associated with the rate of change in weight loss, and/or play a role in regulating the biological variation, in response to energy restriction.

**Objective:**

To quantify targeted c-miRNAs with putative roles in energy metabolism and exercise adaptations following a 16 wk diet and exercise intervention in individuals with large (high responders; HiRes) versus small (low responders; LoRes) losses in body mass.

**Methods:**

From 89 male and female overweight/obese participants who completed the intervention (energy restriction from diet, 250 kcal/d, and exercise, 250 kcal/d), subgroups of HiRes (>10% body mass loss, *n* = 22) and LoRes (<5% body mass loss, *n* = 18) were identified. From resting plasma samples collected after an overnight fast pre and post intervention, RNA was extracted, quantified and reverse transcribed. Thirteen c-miRNA selected *a priori* were analysed using a customised 96-well miScript miRNA PCR Array.

**Results:**

Loss of body mass (-11.0 ± 2.3 kg vs. -3.0 ± 1.3 kg; P<0.01) and fat mass (-11.1 ± 2.6 kg vs. -3.9 ± 1.6 kg; P<0.01) was greater for HiRes than LoRes (P<0.001). Expression of c-miR-935 was higher in LoRes compared to HiRes pre- (~47%; P = 0.025) and post- (~100%; P<0.01) intervention and was the only c-miRNA differentially expressed at baseline between groups. The abundance of c-miR-221-3p and -223-3p increased pre- to post-intervention in both groups (~57–69% and ~25–90%, P<0.05). There was a post-intervention increase in c-miR-140 only in LoRes compared to HiRes (~23%, P = 0.016).

**Conclusion:**

The differential expression and responses of selected c-miRNAs in overweight/obese individuals to an exercise and diet intervention suggests a putative role for these ‘biomarkers’ in the prediction or detection of individual variability to weight loss interventions.

## Introduction

Obesity is the excessive accumulation of fat mass resulting from a chronic imbalance between energy intake and energy expenditure. Common behavioural strategies for weight management and reducing fat mass include dietary energy restriction and increased energy expenditure through physical activity [1]. However, there is a considerable inter-individual variability in weight loss regardless of the weight loss strategy utilised [2–5]. While compliance to prescribed diet and physical activity interventions undoubtedly contributes to the variance in fat loss between individuals, differences in genotype may also be a determinant of the loss of fat during any weight loss intervention [3–5]. Such variability in weight loss response may also, in part, be attributed to epigenetic factors modulated by behavioural/environmental influences [3,6–8].

Small non-coding strands of RNA, microRNAs (miRNAs), are posttranscriptional regulators of messenger RNA (mRNA) that promote mRNA degradation and thereby suppress or inhibit translation [9]. While some miRNAs are highly expressed in various tissues throughout the body, a subset of miRNAs are detectable in circulation (c-miRNAs) and can be transported to other tissues and act in an endocrine manner on targeted cells [3–5,10]. To date, only one study has reported a differential abundance of selected c-miRNAs associated with ‘high’ and ‘low’ responders following an 8-wk energy-restricted diet [4], while others have shown no significant differences before and after a 14-wk weight loss diet despite a 17% loss in body mass [11]. Of note, these studies did not incorporate exercise prescription during the weight loss interventions. Exercise confers multiple beneficial effects on musculoskeletal and cardiorespiratory health and is a well-established modulator of gene expression that can also alter c-miRNA abundance [12,13].

The aim of the current study was to investigate whether the expression of specific c-miRNAs previously shown to be modulated by energy restriction or exercise are ‘predictive’ of the magnitude of weight loss between high’ and ‘low’ responders following a 16 wk diet and exercise intervention. A second aim was to determine whether changes in these c-miRNAs as a result of the weight loss intervention are associated with loss of body mass and changes in glucose tolerance. It was hypothesised there would be a differential abundance of c-miRNAs implicated in the regulation of fat metabolism and exercise adaptation between high and low weight loss responders both before and after the intervention.

## Materials and Methods

### Participants

**Fig 1:**
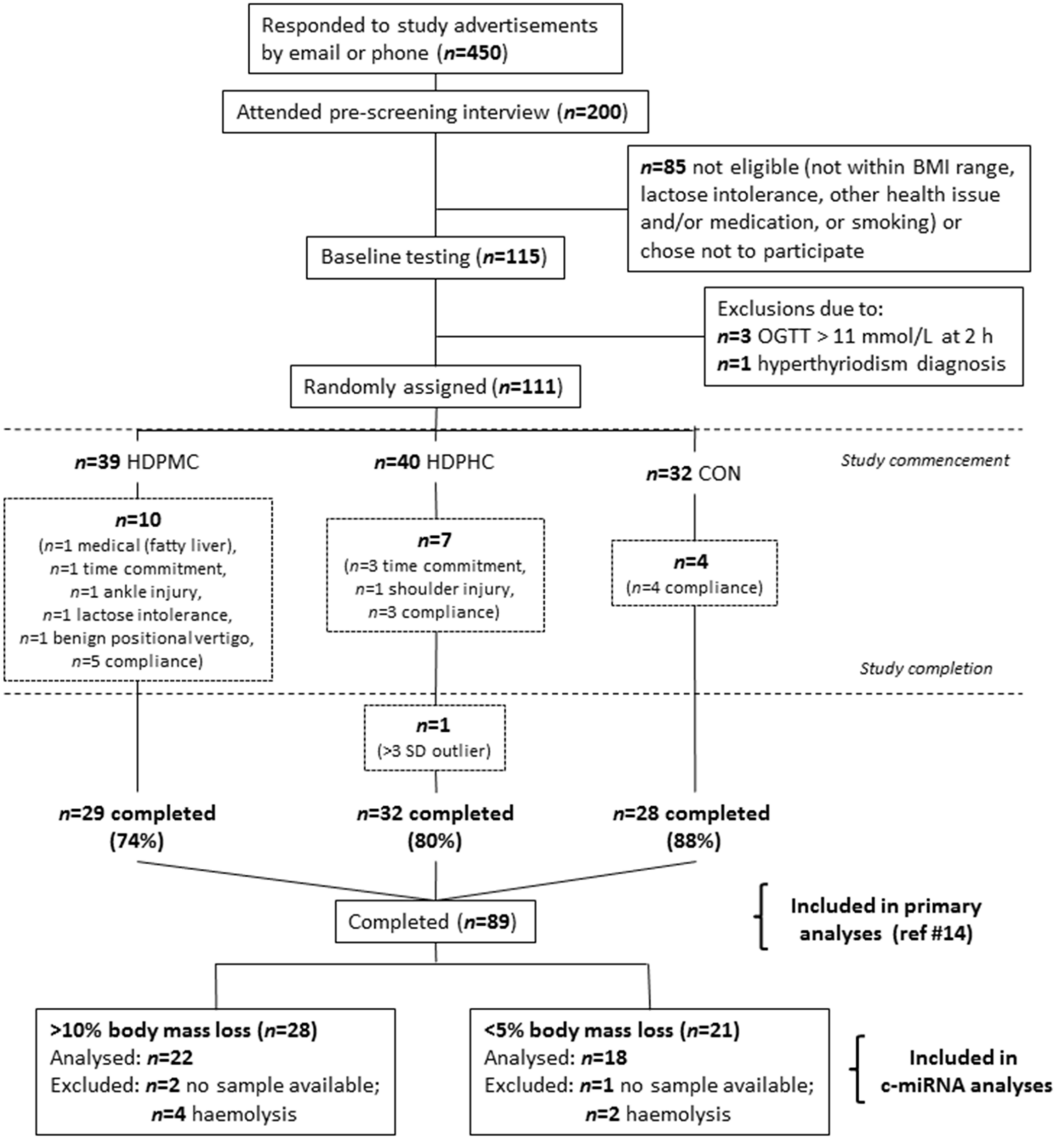
Flowchart of participants throughout the 16 wk diet and exercise intervention and in the subsequent analysis of high (HiRes) and low (LoRes) responders to the intervention. HDPHC, high dairy protein moderate fat; HDPMC, high dairy protein moderate fat; CON, control. Compliance was to the diet and/or the exercise training.

One hundred and eleven males and females aged 35–59 years began a 16-wk weight loss intervention incorporating diet and exercise modification, as previously described [14]. Prior to commencing the intervention, participants had a BMI of 27–40 kg/m^2^ and were sedentary but otherwise apparently healthy. All participants gave written consent to participate in the intervention. This was approved by RMIT Human Research Ethics Committee (76/11). The trial was prospectively registered with the Australian New Zealand Clinical Trials Registry (ACTRN12612000021875). Participants were recruited and participated in the trial between March 2012 and October 2013. Of the initial participant cohort, 89 individuals successfully completed the intervention and were subsequently ranked according to the magnitude of percentage body mass loss pre- to post intervention from highest to lowest (Fig 1), irrespective of dietary group (as described below). From this ranking, participants were divided into quartiles and the criteria for high and low responders was formulated based on the 1^st^ vs 4^th^ quartiles: the upper quartile were individuals who had lost ≥~10% (high responders) and the lower quartile individuals who lost ≤5% (low responders) of initial body mass. There were a total of 49 individuals that were categorized as losing ≤5% or ≥10% of initial body mass after the 16 wk intervention.

### Experimental Design

The experimental design has been described in detail elsewhere [14]. Briefly, participants completed a 16 wk intervention during which an energy deficit of 500 kcal was induced through an energy restricted diet (~250 kcal) and daily exercise (~250 kcal). Participants were stratified according to sex, age, and BMI and then randomly assigned to one of three dietary groups of different macronutrient intake (high dairy protein, high carbohydrate (HDPHC) ~30% protein, 55% CHO, 15% fat; High dairy protein, moderate carbohydrate (HDPMC) ~30% protein, 40% CHO, 30% fat; Low dairy protein, high carbohydrate (control, CON) ~15% protein, 55% CHO, 30% fat). For all groups, energy intake was restricted by 250 kcal/d based on estimated energy balance requirements [15]. Body composition was measured via whole-body dual-energy x-ray absorptiometry (DXA) scans (GE Lunar Prodigy Pro, GE Healthcare; software: Encore 2009, version 12.20.033) before and after the 16 wk intervention. Dietary intake was assessed through analysing 7 day food records in Foodworks^®^ Professional Edition Food Composition database (Version 7.0, Xyris Software Pty Ltd, QLD, Australia) collected in weeks 1, 4, 8, 12 and 16 and averaging intake across the study intervention period.

After an overnight fast participants reported to the laboratory for a DXA scan and fasted blood samples were taken from an antecubital vein. Blood was collected in a non-additive tube (9 mL) and immediately spun at 3000 *g* at 4°C for 10 min before analysis for insulin concentration and a EDTA tube (4 mL) was collected for baseline blood glucose analyses (YSI 2900, YSI Life Sciences, Yellow Springs, OH, USA) as part of the oral glucose tolerance test (OGTT). OGTT’s were conducted before and after the intervention period using a 75 g (300 mL) commercially produced glucose solution (Point of Care Diagnostics, NSW, Australia) consumed within 5 min. Sequential 4 mL blood samples were collected 30, 60, 90 and 120 min post-ingestion. The remaining sample was spun at 3000 g, 4°C, for 10 min and stored at -80°C for later analysis. The homeostasis model assessment (HOMA) score was calculated from glucose and insulin concentrations, as previously described [16].

### Plasma RNA Extraction and Quantification

Plasma samples stored at -80°C were thawed on ice and subsequently centrifuged at 3000 *g* at 4°C for 10 min to pellet cell debris. Thereafter, 1.5 μL of plasma was loaded onto a NanoDrop 1000 spectrophotometer (Thermo Scientific, USA) to determine levels of free haemoglobin [17]. Samples measuring above 0.20 at 414 nm (indicative of elevated free haemoglobin) were excluded from further analysis (*n* = 6). 200 μL of the plasma was then transferred to a 1.5 mL tube and mixed with 1 mL Qiazol lysis reagent (Cat No. 217184; Qiagen, VIC, Australia) and 3.5 μL of miRNeasy Spike-In Control *Caenorhabditis elegans* miR-39 (cel-miR-39; Qiagen, VIC, Australia). 200 μL of chloroform was subsequently added to the samples and vortexed for 15 s before being centrifuged at 12 000 *g* at 4°C for 15 min. The upper aqueous phase (~600 μL) was then transferred to a 2 mL tube and mixed thoroughly with 100% ethanol. Samples were transferred to RNeasy mini spin columns (Qiagen, VIC, Australia) in a 2 mL collection tube and centrifuged at 8 000 *g* for 15 s at room temperature. After washing and centrifuging with RWT and RPE buffers (Qiagen, VIC, Australia), columns were washed a final time with 80% ethanol. Columns were then centrifuged with lids open to dry any residual ethanol as per the manufactures directions. For RNA elution, columns were placed in a new 1.5 mL collection tube and 14 μL of RNase-free water was added directly to the spin column membrane and centrifuged for 1 min at 16 000 *g* (maximum speed) at room temperature. Extracted RNA was quantified using the NanoDrop 1000 spectrophotometer and measuring absorbance at 260 nm and 280 nm and equilibrated with RNase-free water for subsequent cDNA synthesis.

### Reverse Transcription (RT) and Pre-Amplification

50 ng RNA was reverse transcribed using a miScript II RT Kit (Cat. No. 218160, Qiagen, Melbourne, Australia) in a BioRad thermal cycler (BioRad, Australia) according to the manufacturer’s protocol. The resulting cDNA was then diluted in RNase-free water and pre-amplified using a miScript PreAMP PCR Kit (Cat No. 331452; Qiagen, Australia) according to the manufacturer’s specifications. Pre-amplified cDNA was then diluted in RNase-free water and stored at -20°C.

### Real-Time PCR

Quantification of miRNAs was performed on a Qiagen customized 96-well miScript miRNA PCR Array (Custom Catalogue Number: CMIHS02269). The array contained positive and reverse transcription controls, and 13 miRNAs selected *a priori* from previous studies showing changes following exercise, dietary intervention and/ or weight loss including: hsa-miR-21-5p, hsa-miR-126-3p, hsa-miR-140-5p, hsa-miR-142-3p, hsa-miR-148a-5p, hsa-miR-148b-5p, hsa-miR-199a-3p, hsa-miR-221-3p, hsa-miR-223-3p, hsa-miR-423-5p, hsa-miR-589-3p, hsa-miR-874-5p and hsa-miR-935. PCR arrays were run using a miScript SYBR Green PCR Kit (218073; Qiagen, VIC Australia) with microRNA abundance normalized to cel-miR-39-3p abundance, and expression was not different at either time point or between groups (P = 0.77). The 2^ΔΔ^CT method of relative quantification was used to calculate the relative abundance of miRNAs in plasma [18]. Where the relative abundance of miRNAs in plasma were >3 SD from the mean, measures were excluded from analysis at both time points (i.e. pre and post).

### Selection of Predicted miRNA Targets

The Targetscan bioinformatics algorithm (Version 6.2, www.targetscan.org) was used for target prediction of miRNAs altered between high and low responders or pre to post intervention for those that were determined to be statistically significant (P<0.05).

### Statistical analysis

To estimate whether differences between time points were statistically significant, a linear mixed model with an AR1 covariance matrix was used for each outcome measure. The interaction between time × group was included to allow the groups to change differently over time and to obtain least significant difference (LSD) post hoc comparisons of group within time. As this was an exploratory study, no adjustments were made for multiple comparisons in order to avoid type II errors [19]. Independent sample t-tests were used to assess baseline participant characteristics pre intervention and baseline differences in c-miRNA expression where main effects were observed. Linear regression analysis was performed to assess the relationship between baseline participant characteristics and baseline c-miRNA abundance, and between changes in participant characteristics and c-miRNA abundance pre- and post-intervention. Data are mean ± standard deviation (SD) and were analysed using SPSS (Version 20.0) with significance set at P<0.05.

## Results

### Participant characteristics

The mean age of participants was 47 ± 6 y. Forty-nine individuals lost ≤5% or ≥10% of their initial body mass after the 16 wk intervention. Participant characteristics pre and post intervention are listed in Table 1.

**Table 1:** Participant characteristics measured pre and post intervention of High (HiRes) and low (LoRes) responders to the 16 week diet and exercise intervention.

	Low responders (*n* = 18)		High responders (*n* = 22)		P values		
	Pre	Post	Pre	Post	Group	Time	Interaction
Age (years)	46.3 ± 5.7	-	48.2 ± 6.2	-	-	-	-
Body mass (kg)	94.4 ± 11.7	92.3 ± 12.0^#^	89.9 ± 14.2	82.8 ± 12.1^#^*	P = 0.04	P<0.01	P<0. 01
Body Mass Index (kg/m^2^)	33.6 ± 4.7	32.5 ± 4.5^#^	31.9 ± 3.7	27.9 ± 3.3^#^*	P = 0.02	P<0.001	P<0.001
Fat mass (kg)	41.2 ± 11.2	37.4 ± 12.0^#^	37.5 ± 8.7	26.4 ± 8.3^#^*	P = 0.03	P<0.01	P<0.001
Lean mass (kg)	50.2 ± 8.2	51.1 ± 8.1^#^	49.3 ± 9.5	49.5 ± 9.7	NS	P = 0.05	NS
Fasting blood glucose concentration (mmol/L)	5.3 ± 0.5	5.3 ± 0.5	5.8 ± 0.7*	5.4 ± 0.6^**#**^	NS	P = 0.02	P<0.01
Oral glucose tolerance test AUC (AU)	210 ± 104	159 ± 78	316 ± 153*	186 ± 88^#^	P = 0.04	P<0.001	P = 0.05

Data are mean ± SD; P<0.05:

^#^ difference between time points within condition

* difference between groups within time points

Nine participants were excluded from analysis: six (*n* = 4 HiRes/*n* = 2 LoRes) through blood sample haemolysis and three with missing samples (*n* = 2 HiRes/*n* = 1 LoRes) (Fig 1). Therefore, 18 LoRes and 22 HiRes participants were included in the final analysis and each diet intervention was represented in both the HiRes (*n* = 8 HPMC, *n* = 5 HPHC, *n* = 9 CON) and LoRes (*n* = 6 HPMC, *n* = 9 HPHC, *n* = 3 CON) groups.

### Plasma c-miRNA

**Fig 2:**
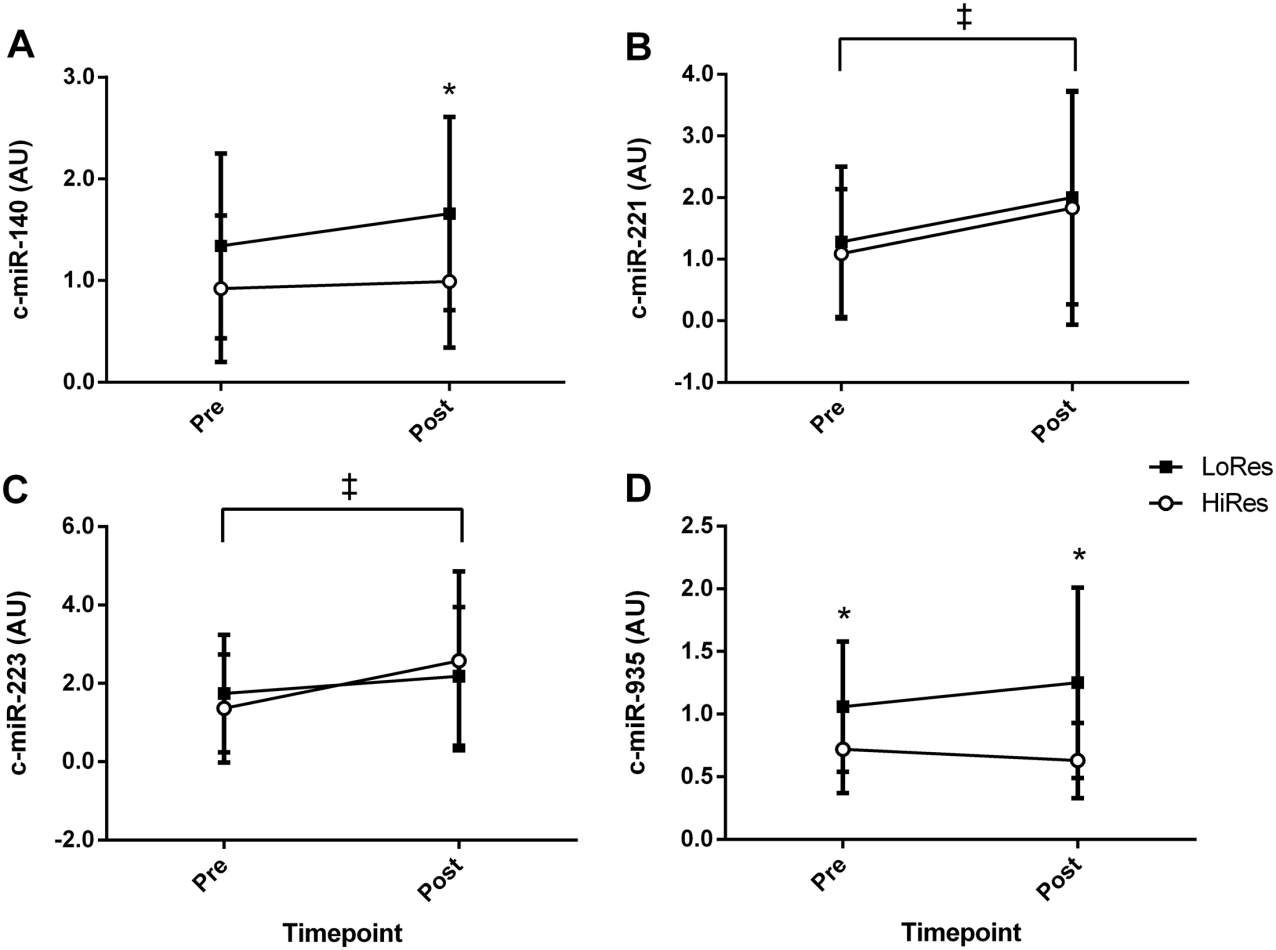
Plasma c-miR abundance prior to and after a 16 week diet and exercise weight loss intervention for high responders (HiRes, *n* = 22) and low responders (LoRes, *n* = 18) A: c-miR-140, B: c-miR-221; C: c-miR-223, and D: c-miR-935; data are mean ± SD; P<0.05: ‡ difference pre vs post, * difference HiRes vs LoRes.

The c-miR-221-3p and -223-3p abundance increased across time from pre- to post-intervention in both groups (57–69% and 25–90%, P = 0.04 and P = 0.05, respectively; Fig 2B and 2C). There was a significant effect for time for c-miR-223 (P = 0.05) as a result of the increase in expression above baseline in HiRes after the intervention (Fig 2C; P = 0.038). A group difference was observed for c-miR-935 (P = 0.001), where c-miR-935 was the only microRNA differentially expressed at baseline being higher in LoRes than HiRes (47%; P = 0.046, Fig 2D, Table 2). Such a difference in c-miR-935 was still evident at the conclusion of the 16 wk intervention period (100%; P<0.001). A group difference for c-miR-140 was evident (P = 0.016) and post hoc tests showed no difference between responders at baseline for c-miR-140 but an increase in expression in LoRes (23%) compared to HiRes post-intervention (8%, P = 0.014). There were modest effects of the 16 wk weight loss intervention on the abundance of other c-miRNA targets (Table 2). Raw plasma c-miRNA Ct data can be found in the S2 Dataset.

**Table 2:** Plasma c-miRNA abundance at baseline (pre-intervention) and the relative fold change in abundance after a 16 wk diet and exercise intervention in low and high responders for weight loss.

c-miRNA	Pre-intervention			Relative fold change		
	Low responders (*n* = 18)	High responders (*n* = 22)	P-value	Low responders (*n* = 18)	High responders (*n* = 22)	P value
miR-21-5p	2.47 ± 2.44	1.94 ± 2.30	0.96	0.28 ± 0.88	-0.05 ± 1.11	0.30
miR-126-3p	1.41 ± 1.10	1.16 ± 0.85	0.41	0.27 ± 1.16	0.25 ± 1.24	0.84
miR-140-5p	1.34 ± 0.91	0.92 ± 0.72	0.12	0.23 ± 0.66	0.07 ± 1.04	0.44
miR-142-3p	1.42 ± 1.03	0.98 ± 0.75	0.12	-0.02 ± 0.95	0.22 ± 1.08	0.54
miR-148a-5p	1.15 ± 0.76	0.92 ± 0.67	0.35	-0.10 ± 1.11	-0.02 ± 0.67	0.75
miR-148b-5p	1.39 ± 1.26	0.82 ± 0.63	0.08	-0.03 ± 1.08	0.97 ± 2.76	0.34
miR-199a-3p	2.50 ± 2.45	1.39 ± 1.42	0.08	0.19 ± 1.41	0.72 ± 1.87	0.60
miR-221-3p	1.28 ± 1.22	1.09 ± 1.06	0.62	0.51 ± 1.43	0.63 ± 1.97	0.97
miR-223-3p	1.75 ± 1.50	1.36 ± 1.38	0.42	0.25 ± 1.48	0.82 ± 1.77	0.34
miR-423-5p	1.11 ± 0.58	1.18 ± 0.94	0.80	0.14 ± 0.76	0.65 ± 1.40	0.68
miR-589-3p	1.37 ± 1.03	0.88 ± 1.22	0.20	0.04 ± 1.42	0.22 ± 1.22	0.78
miR-874-5p	1.12 ± 0.79	0.81 ± 0.54	0.17	0.21 ± 1.10	0.18 ± 1.33	0.84
miR-935	1.06 ± 0.52	0.72 ± 0.35	0.03	0.17 ± 0.74	-0.12 ± 0.59	0.18

### Target prediction of altered c-miRNAs

TargetScan identified 345 predicted targets for c-miR-140, 448 for c-miR-221, 310 for c-miR-223 and 263 for c-miR-935. Of these, predicted targets related to obesity, diabetes, substrate metabolism and physiological adaptation responses to exercise (e.g. mitochondrial biogenesis, muscle hypertrophy) were further classified (Table 3).

**Table 3:** Selected target mRNAs of microRNAs showing altered expression patterns (P<0.05) between high and low responders pre and post energy-restricted diet and exercise intervention. Target mRNAs have been previously implicated in substrate (fat) metabolism, obesity, diabetes and exercise adaptation responses.

c-miRNA	Sequence (source: mirbase.org)	TargetScan algorithm	Description (source: www.ncbi.nlm.nih.gov)
hsa-mir-140	UGUGUCUCUCUCUGUGUCCUGCCAGUGGUUUUACCCUAUGGUAGGUUACGUCAUGCUGUUCUACCACAGGGUAGAACCACGGACAGGAUACCGGGGCACC	ARL15	Expressed in insulin-responsive tissues including adipose tissue and skeletal muscle; implicated in insulin signaling and insulin-stimulated glucose transport [20]
		CROT	Involved in lipid metabolism and fatty acid beta-oxidation
		CRTC3	May induce mitochondrial biogenesis and attenuate catecholamine signaling in adipose tissue
		FNDC5	Gene encodes a secreted protein released from muscle cells during exercise implicated in the development of brown fat
		SOCS7	Proposed to regulate glucose homeostasis [21]
		STRADB	Component of a kinase protein complex regulating energy-generating metabolism
hsa-mir-221	UGAACAUCCAGGUCUGGGGCAUGAACCUGGCAUACAAUGUAGAUUUCUGUGUUCGUUAGGCAACAGCUACAUUGUCUGCUGGGUUUCAGGCUACCUGGAAACAUGUUCUC	CYP7A1	Gene encodes a member of the cytochrome P450 superfamily of enzymes catalysing the synthesis of cholesterol and other lipids
		PIK3R1	Involved in the metabolic actions of insulin
		SOCS7	Proposed to regulate glucose homeostasis [21]
hsa-mir-223	CCUGGCCUCCUGCAGUGCCACGCUCCGUGUAUUUGACAAGCUGAGUUGGACACUCCAUGUGGUAGAGUGUCAGUUUGUCAAAUACCCCAAGUGCGGCACAUGCUUACCAG	HDAC4	Involved in histone acetylation and chromatin remodelling with a potential role in GLUT4 transcription [22]
		IGF1R	Receptor binding insulin-like growth factor responsible for the activation of the IGF-signalling cascade
		PLA2G6	Catalyses the release of fatty acids from phospholipids
		SORBS1	Involved in the signaling and stimulation of insulin
hsa-mir-935	GGCGGGGGCGCGGGCGGCAGUGGCGGGACGGCCCCUCGGCCAUCCUCCGUCUGCCCAGUUACCGCUUCCGUACCGCCGCCGCUCCCGCU	HMGB1	Putative involvement in the maintenance of chronic low-grade inflammation associated with increased adiposity [23]
		MEF2D	Involved in the control of muscle cell differentiation and development
		PHIP	Modulates insulin signaling
		PPARGC1B	Stimulates the activity of several transcription factors and nuclear receptors involved in fat oxidation, non-oxidative glucose metabolism, and the regulation of energy expenditure.

Key: ARL15, ADP-ribosylation factor-like 15; CROT, carnitine O-octanoyltransferase; CRTC3, CREB regulated transcription coactivator 3; CYP7A1, cytochrome P450, family 7, subfamily A, polypeptide 1; FNDC5, fibronectin type III domain containing 5; HDAC4, histone deacetylase 4; HMGB1, high mobility group box 1; IGF1R, insulin-like growth factor 1 receptor; MEF2D, myocyte enhancer factor 2D; PIK3R1, phosphoinositide-3-kinase, regulatory subunit 1 (alpha); PHIP, pleckstrin homology domain interacting protein; PLA2G6, phospholipase A2, group VI (cytosolic, calcium-independent); PPARGC1B, peroxisome proliferator-activated receptor gamma, coactivator 1 beta; SOCS7, suppressor of cytokine signaling 7; SORBS1, sorbin and SH3 domain containing 1; STRADB, STE20-related kinase adaptor beta.

### Anthropometric, diet and changes in aerobic fitness

There were no differences in body mass, BMI and fat mass between groups at baseline (Table 1). The HiRes participants lost 8 kg more body mass (P<0.01) and 7.2 kg more fat mass (P = 0.03), than LoRes respectively, with significant group × time interactions (both P<0.01). A corresponding decrease in BMI was also evident for HiRes compared to LoRes following the 16 wk intervention period (P = 0.02) where an interaction effect was also observed (P<0.001).

Using the Mifflin St Jeor equation [15] for sedentary individuals, the energy requirements of HiRes and LoRes were 2081 ± 352 kcal/d and 2174 ± 202 kcal/d, respectively. Dietary analysis of individual food diaries obtained during the diet intervention showed the reported energy intake was not different between groups (HiRes: 1622 ± 262 kcal/d (*n* = 18) vs. LoRes: 1606 ± 136 kcal/d (*n* = 15), P = 0.84). Pre-intervention peak aerobic power (VO_2_peak) did not differ between groups (HiRes: 2.21 ± 0.68 L/min; LoRes: 2.20 ± 0.56 L/min, P = 0.97), and was significantly increased (P<0.001) by the same magnitude in response to exercise training (HiRes: 0.27 ± 0.19 L/min, LoRes: 0.25 ± 0.18 L/min, P = 0.70).

### Blood parameters

At baseline, fasting blood glucose was higher in HiRes than LoRes (Table 1, P = 0.008), with a significantly greater decrease in blood glucose in HiRes over the 16 wk intervention than LoRes (P<0.001). The area under the curve (AUC) from the OGTT was also greater in HiRes compared with LoRes pre-intervention (Table 1, P = 0.006). The 16 wk intervention resulted in a reduction in glucose AUC (-91 ± 117; P<0.01) across both groups that was significant for HiRes (P<0.001), but not the LoRes group (P = 0.07). A group × time interaction effect was observed for insulin concentration (P = 0.05). Insulin concentrations were not different between groups at baseline (HiRes: 8.1 ± 5.4 mIU/mL vs. LoRes: 7.3 ± 6.0 mIU/mL, P = 0.60) but decreased significantly over the 16 wk intervention in the HiRes group only (HiRes: -2.9 ± 4.1 mIU/mL, P<0.001; LoRes: -1.2 ± 3.9 mIU/mL; P = 0.51). No differences in HOMA index were observed between groups despite a decrease across time (HiRes, Pre: 2.15 ± 1.69, Post: 1.21 ± 0.75; LoRes, Pre: 1.80 ± 1.63, Post: 1.61 ± 1.27 P = 0.003) with a significant group × time interaction (P = 0.045).

### Relationships between c-miRNAs and anthropometric measures

Regression equations were used to show the abundance of several baseline c-miRNA’s were weakly associated with pre-intervention body mass including c-miR-221 (P = 0.02, R^2^ = 0.15), c-miR-21 (P = 0.01, R^2^ = 0.16) and c-miR-126 (P = 0.05, R^2^ = 0.10). These weak associations explained less than 20% of the variance between baseline c-miRNA abundance and pre-intervention body mass. There were no relationships between baseline c-miRNA abundance and pre-intervention fat mass, lean mass or BMI, while a weak relationship was found between baseline c-miR-140 and age (P = 0.04, R^2^ = 0.11). There were no significant relationships between the change in c-miRNA abundance and the changes in total, fat or lean masses as a result of the 16 wk diet and exercise intervention.

## Discussion

This is the first study to characterise changes in targeted c-miRNAs in a cohort of high and low responders to a chronic diet- and exercise intervention in overweight/obese individuals. We report altered expression of c-miRNA-140 and -935 in human plasma that were associated with differences in the magnitude of change in body mass, along with differential changes in the expression of c-miRNA-221 and -223 pre- to post-intervention. These findings provide novel information that may link these c-miRNA to the beneficial effects of exercise- and diet-induced energy restriction for weight loss.

Diet- and exercise-based weight loss interventions result in large variability in responses between individuals that is often under-reported [24]. The expression profile of particular c-miRNAs have emerged as promising ‘biomarkers’ for certain disease states, raising the possibility that the regulation of c-miRNA may be involved in the heterogeneity in body composition changes between individuals during interventions aimed at losing body mass. By utilising previous studies that had demonstrated robust changes in c-miRNA following exercise, energy restriction or surgical interventions with putative roles in weight loss, we identified 13 c-miRNA for analysis [4,11,25,26]. Results from the current study show increased levels of c-miR-935 in the LoRes compared to HiRes at baseline and increases in c-miR-140, -221 and -223 abundance following a 16 wk weight-loss intervention. We are the first to report a selective increase in c-miR-140 abundance in participants with small (LoRes) compared to those with a larger change in body mass (HiRes) in response to an energy restricted diet and exercise weight-loss intervention. Increased basal c-miR-140 abundance has been reported in obese/morbidly obese groups and was markedly reduced after bariatric surgery [11], although its expression was unchanged following a diet-only weight loss intervention [11]. Furthermore, higher c-miR-140 abundance has been reported in individuals with a high (BMI ≥ 40 kg/m^2^) compared to normal (BMI≤ 30 kg/m^2^) body mass [11].

A putative target of c-miR-140 is the muscle integral membrane protein FNDC5. The hormone irisin, the proteolytically processed product of the FNDC5 gene, is secreted by skeletal muscle cells in response to exercise where it has been shown to circulate to white adipose tissue and stimulate ‘beige’ fat development [27]. It is possible the higher c-miR-140 expression post-intervention only in LoRes downregulates FNDC5 activity and subsequently reduces its ability to promote weight loss through reduced energy expenditure.

The increased expression of c-miR-935 in LoRes compared to the HiRes group (Fig 2D) is in agreement with previous findings of higher c-miR-935 abundance at baseline in a group of non-responders (<5% body weight) to a low energy (~850 kcal/d) diet-only weight-loss intervention [4]. Using the TargetScan algorithm, several predicted targets of c-miR-935 were identified with purported roles in various exercise-mediated adaptations and energy metabolism (Table 3). It is currently unknown if this disparity in baseline c-miR-935 abundance between the HiRes and LoRes groups up- or down-regulates the expression of these targets in a tissue-specific manner (i.e. adipose tissue, skeletal muscle, etc.). However, of note was that the differential expression persisted after the intervention. Regardless, our findings support those of Milagro and colleagues [4] and provide further evidence that c-miR-935 may act as a ‘biomarker’ to differentiate between individuals with high and low responses in weight loss.

Recent evidence from overweight and obese humans suggests c-miR-223 as a potential biomarker for obesity [28,29]. Indeed, reduced c-miR-223 levels have been observed in morbidly obese (BMI >40 kg/m^2^) and obese (BMI 30–39.9 kg/m^2^) compared to normal-overweight individuals (BMI 20–29.9 kg/m^2^) [28]. Moreover, c-miR-223 abundance has been shown to increase after a 12 wk unstructured dietary and exercise intervention in obese and overweight participants [29]. These results indicate that increases in c-miR-223 are associated with diet and exercise interventions that result in loss of body mass. The increases in c-miR-223 with weight loss is in agreement with our findings as we report increased c-miR-223 and -221 abundance post-intervention in both HiRes and LoRes groups. Previous reports of c-miR-221 responses to dietary or exercise interventions are equivocal, with a single study showing a decrease in c-miR-221 three days after a bout of resistance exercise [26] while another reported increased c-miR-221 abundance after bariatric surgery and subsequent weight loss but not dietary-induced weight loss [11]. Thus, the increase in c-miR-221 abundance across the 16 wk intervention in both LoRes and HiRes (Fig 2B) indicates these changes are most likely related to the exercise stimulus rather than being diet-induced.

Although participants were grouped according to a loss of body mass, glucose tolerance was also different between groups at baseline, with higher fasting glucose concentration and a greater area under the curve for OGTT in HiRes. Therefore, it is plausible the reduced glucose sensitivity at baseline conferred greater likelihood of observing a change in sensitivity and subsequent weight loss, and that enhanced glucose disposal/glycaemic stability in response to the exercise and diet intervention may be a hallmark of HiRes. While the c-miR-126 has been implicated as a biomarker for impaired fasting glucose and glucose tolerance [30], we found no difference in c-miR-126 abundance between HiRes and LoRes groups, suggesting any potential for c-miR-126 modulation with impaired glucose metabolism may be limited to a pre-diabetic population [30]. Nonetheless, glucose tolerance may still be an underlying determinant for predicting a weight loss responder to an exercise and diet intervention.

The divergent loss of body mass between HiRes and LoRes does not appear to be related to the adaptive responses to endurance exercise as no differences were observed post-intervention in aerobic fitness (VO_2_peak) between the two groups. Further, only weak correlations were found between baseline body mass, BMI, or fat mass measures and selected c-miRNA abundance in the current study and no significant correlations between changes in anthropometric measures and changes in c-miRNA abundance were observed. These results contrast those of Milagro and colleagues [4] who reported strong correlations between several miRNA’s measured in peripheral blood mononuclear cells and the magnitude of weight loss (*n* = 10). However, a strength of the current study was the larger participant cohort (*n* = 40) to provide a greater representation of the overweight/obese population. The inclusion of exercise in addition to dietary energy restriction to generate the overall energy deficit may also have contributed to disparity in results between studies.

A limitation of the present work, and other studies that have investigated c-miRNA responses in humans to exercise and diet interventions, is the lack of concomitant sampling of tissues (i.e. skeletal muscle and adipose tissue) in which c-miRNAs may originate or target in order to mediate specific cellular processes regulating weight loss. Simultaneous measurements of muscle and adipose tissues would provide an integrated approach to better understand the interplay and ‘cross talk’ between different tissues to a given stimulus, such as a weight loss intervention. The time-course of appearance and disappearance of individual c-miRNAs following an exercise and diet intervention also remains to be established and may vary according to the length of intervention and the time in which samples were obtained.

In conclusion, this is the first study to report the expression profile of selected c-miRNAs following a structured exercise and diet weight loss intervention. This study provides new information demonstrating that the abundance of c-miRNA -221 and -223 are modulated with exercise and diet, and that c-miRNA -935 and 140 are differentially expressed between high and low responders before and after a chronic weight loss intervention. Our findings reveal the potential for c-miRNAs to act as ‘biomarkers’ regulating the magnitude of weight loss to an exercise and diet intervention between different individuals. *In vitro* mechanistic studies in cell culture and animal knockout models are required to validate the regulation of target proteins by their specific c-miRNAs. Further research using larger participant cohorts over longer interventions will be important for determining whether c-miRNAs regulate cellular mechanisms controlling weight loss and therefore become accurate biomarkers for obesity risk and development.

## Supporting Information

S1 Dataset Raw participant characteristics, blood markers and aerobic capacity measured pre and post a 16 wk diet and exercise intervention in low (LoRes) and high (HiRes) responders for weight loss. (XLSX)

S2 Dataset Raw Ct data for the 13 plasma c-miRNA abundance measured pre and post a 16 wk diet and exercise intervention in low (LoRes) and high (HiRes) responders for weight loss. (XLSX)
